# Optimization of CNC milling parameters for YXR-7 tool steel using fuzzy MARCOS: A multi-response approach to improve machining productivity

**DOI:** 10.1371/journal.pone.0352316

**Published:** 2026-07-29

**Authors:** Adooru L. N. Arunkumar, Sunil Kumar, Krishnadas Narayanan Nampoothiri, Abhishek Jha, Ankur Jaiswal

**Affiliations:** 1 Department of Mechanical Engineering, Amrita School of Engineering, Amrita Vishwa Vidyapeetham, Chennai, India; 2 Department of Mechanical Engineering, CVR College of Engineering, Hyderabad, India; 3 Department of Mechanical Engineering, SRM University-AP, Amaravati, Mangalagiri, Andhra Pradesh, India; 4 Manipal Institute of Technology, Manipal Academy of Higher Education, Manipal, India; Ramaiah Institute of Technology, INDIA

## Abstract

This study presents an integrated optimisation method for CNC milling of heat-treated YXR7 tool steel using carbide cutting inserts under varying lubrication and process parameters. A full factorial experimental design comprising 27 runs was employed to assess the influence of depth of cut (*d*_*c*_), feed per tooth (*f*_*t*_), cutting speed (*C*_*s*_), and nano-cutting fluid (*C*_*f*_) on critical performance responses such as surface roughness (*Ra*), material removal rate (*MRR*), and tool wear rate (*TWR*). An advanced modelling through regression and ANOVA showed complex interactive and non-linear effects among process parameters. To effectively navigate these interdependencies, a novel hybrid decision-making model combining the Full Consistency Method (FUCOM) and fuzzy-MARCOS was employed. This multi-criteria decision-making (MCDM) method was described for uncertainties in machining performance and successfully ranked experimental alternatives based on their proximity to ideal performance. The optimal configuration (Experiment 21) accomplished a superior balance across all criteria, notably achieving a low surface roughness (Ra ≈ 0.42 µm) and *TWR* (~0.148 mm³/min) while maintaining a high *MRR* (~109.4 mm³/min). The proposed fuzzy-FUCOM-MARCOS method reveals high robustness, adaptability, and decision reliability, contributing a valuable strategy for precision machining of hard-to-cut steels. This work bridges experimental understandings with intelligent optimisation, fostering sustainable and high-performance manufacturing practices in the tooling industry.

## 1. Introduction

Achieving precision and productivity in manufacturing demands efficient milling operations, particularly when machining hard alloys like YXR7 tool steel, which are increasingly in demand across industries. Traditional techniques such as electrical discharge machining (EDM) are progressively being replaced by hard milling due to its advantages in productivity and environmental sustainability [1,2]. Existing literature supports that optimising cutting parameters plays a vital role in prompting surface integrity and chip morphology, both crucial for confirming the desired mechanical properties in finished components [1,3]. Research on Vanadis® 8 tool steel additionally highlights that the inclusion of carbides improves the microstructure, improving overall milling performance [4]. Additionally, advanced methods such as helical milling have confirmed improvements in surface finish and tool longevity, underlining the significance of process planning in the machining of hard alloys [2].

Precisely, the optimisation of CNC milling parameters such as cutting speed, feed rate, depth of cut, and the use of nano-cutting fluids can significantly boost manufacturing efficiency and product quality. Prior studies have shown that such optimisation leads to reduced tool wear and machining time, while increasing the material removal rate (MRR) and improving surface finish [5,6]. For example, hybrid optimisation techniques like the Kriging model with NSGA-II have generated favourable trade-offs between cutting forces and MRR under hard milling conditions [7]. Nano-cutting fluids offer larger cooling and lubrication, enhancing machining products [7]. Algorithms such as Taguchi and tool life curves have also been used to identify optimal parameter sets, significantly decreasing deviations in geometric dimensions and tool wear during the machining of materials like Inconel 718 and Ti alloys at speeds up to 80 m/min [8,9].

Despite significant advancements in machining parameter optimisation, conventional methods such as Taguchi methods, Grey Relational Analysis (GRA), and evolutionary algorithms like NSGA-II show notable limitations when applied to multi-response, complex machining problems. These methods are generally deterministic in nature and often struggle to efficiently handle the imprecision, uncertainty, and conflicting objectives essential in real-world manufacturing environments. For instance, Taguchi-based optimisation primarily focuses on single-response scenarios, whereas GRA may oversimplify the interrelations among responses. Similarly, although NSGA-II is powerful for multi-objective optimisation, it requires substantial computational effort and does not explicitly incorporate subjective decision-making preferences, which are critical in industrial applications.

To address these challenges, modern research has gradually focused on advanced multi-criteria decision-making techniques integrated with fuzzy logic to manage uncertainty and ambiguity in machining data. The FUCOM provides a structured method for determining criteria weights using the least number of pairwise comparisons while ensuring maximum consistency and reduced redundancy [10]. This increases the reliability of decision-making compared to the traditional weighting approach. Simultaneously, the fuzzy-MARCOS method estimates alternatives based on their relative closeness to ideal and anti-ideal solutions within a fuzzy approach, thus effectively capturing uncertainty and linguistic uncertainty in performance evaluation [11]. The integration of FUCOM with fuzzy-MARCOS suggests a robust hybrid optimisation approach that simultaneously addresses consistency in weighting and uncertainty in ranking, which makes it highly suitable for multi-response optimisation in advanced machining processes.

Unlike conventional MARCOS-based optimisation studies, which typically use subjective weighting structures and deterministic input data, the present study integrates the FUCOM method to determine consistent and reliable criteria weights with minimal pairwise comparisons. Furthermore, the integration of fuzzy logic within the MARCOS method enables effective handling of uncertainty and imprecision in machining responses. This integrated FUCOM–fuzzy MARCOS approach thus provides improved consistency, robustness, and decision accuracy compared to previously reported MARCOS applications in machining optimisation. Their integration produces a robust framework suitable for complex machining scenarios. Recent studies confirm the success of such methods in EDM and sustainable machining conditions [12–15]. Accordingly, this study suggests a FUCOM-integrated fuzzy-MARCOS framework to optimise CNC milling parameters for YXR7 tool steel, focusing on tool wear, surface roughness, and MRR to enhance the performance and sustainability.

However, existing optimisation frameworks generally depend on single-objective approaches and fail to effectively address uncertainty and conflicting responses in machining. Additionally, limited studies have investigated the integration of FUCOM with fuzzy-MARCOS in the milling of hard tool steels, particularly under nanofluid environments. To address these gaps, the present study proposes a comprehensive optimisation approach with the following major contributions, i.e., (i) development of a FUCOM–fuzzy MARCOS hybrid method for CNC milling parameter optimisation, (ii) integration of nanofluid lubrication to multi-response optimisation, (iii) experimental validation using a full factorial design of experiment and (iv) establishment of a robust decision-support system for industrial machining applications.

## 2. Methodology

### 2.1. Experimental setup

Heat-treated YXR-7 tool steel (sample) of hardness 55–60 HRC was chosen for its proven durability in tooling applications. It consists of 5.5% Mo, 4.7% Cr, 1.3% W, 1.3% V, 0.8% C and the rest Fe. The increased hardness and carbide-rich microstructure substantially affect machining behaviour, particularly in terms of cutting forces, tool wear, and surface finish. These properties are critical in understanding the machining response and confirming the repeatability of the present study. Cylindrical samples (60 mm diameter × 50 mm length) were machined using ISO-513 H-type tungsten carbide inserts (0° relief, 17° rake) under symmetrical milling and diverse lubrication conditions (Table 1). This insert is suitable for machining hardened materials. The carbide insert was uncoated, assisting direct assessment of tool wear mechanisms without the interference of coating effects, thus ensuring more reliable interpretation of machining performance. Milling was performed on a CNC machine (LOKESH TL model), systematically varying four parameters: depth of cut (*d*_*c*_), feed per tooth (*f*_*t*_), cutting speed (*C*_*s*_), and nano-cutting fluid (*C*_*f*_). The nanoparticles (with 99% purity, average particle size of 90 nm, and concentration of 1.2 wt%) are mixed with both the lubricants (lubrication oil and coolant water), while the base lubricant meets NSF H1 standards. Surface roughness (Ra), material removal rate (MRR), and tool wear rate (TWR) were the primary characteristics. Surface roughness was recorded by a SURFTEST SJ-210 (Mitutoyo, India), while MRR and TWR were computed by the standard formulas [15]:

**Table 1:** Metal cutting parameters and their ranges.

Metal cutting parameters	Units	Levels		
		L_1_	L_2_	L_3_
Depth of cut	mm	1.5	2	2.5
Feed per tooth	mm/tooth	0.2	0.3	0.4
Cutting speed	m/min	575	675	775
Nano-cutting fluid	–	S-Oil*	O-Al_2_O_3_**	W-Al_2_O_3_***

Note: Different lubricants

*S-Oil – Soluble oil

**O-Al_2_O_3_ – Nano particles of Al_2_O_3_ mixed with lubrication oil

***W-Al_2_O_3_ – Nano particles of Al_2_O_3_ mixed with coolant water


$$\begin{array}{c} MRR=\frac{W_{ji}-W_{if}}{\rho \times t} \end{array}$$ (1)

Where: $W_{ji}$ - initial weight of the workpiece before machining, $W_{if}$ - final weight of the workpiece after machining, ρ - density of the workpiece material, t - machining time


$$\begin{array}{c} TWR=\frac{W_{ti}-W_{tf}}{\rho \times t} \end{array}$$ (2)

Where: $W_{ti}$ - initial weight of the tool before machining, $W_{tf}$ - final weight of the tool after machining.

**Fig 1:**
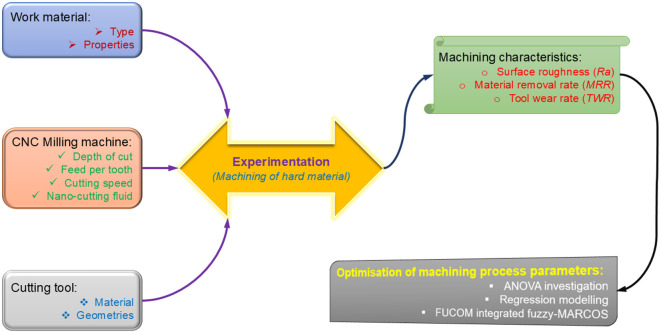
Evaluation and optimisation of machining process parameters.

These formulations were used to quantify the volumetric material removed from the workpiece and the tool wear during the machining process. Each experiment was repeated three times, and the average values of surface roughness, material removal rate and tool wear rate were considered for analysis. Microsoft Excel was used to implement the FUCOM integrated fuzzy-MARCOS model. The complete experimental setup and intelligent system employed in this study are illustrated in Fig 1.

### 2.2. Fuzzification function

In this study, a specific form of the triangular fuzzy number (TFN) was employed, as proposed by Kundu et al. [16]. A TFN is typically denoted as:


$$\begin{array}{c} \tilde{A}=\;(l,\;m,\;n) \end{array}$$ (3)

where *m* represents the modal (most probable) value, while *l* and *n* denote the lower and upper bounds, respectively. This formulation effectively captures uncertainty and vagueness in expert judgments and is widely used in fuzzy logic applications.

The triangular fuzzy number is particularly well-suited for MCDM contexts, where imprecision in human judgment is prevalent. Its application has been extensively adopted in various MCDM methodologies, as supported by recent literature.

Furthermore, the basic arithmetic operations between two TFNs $\tilde{A_{\text{1}}}=\;\left(l_{\text{1}},\;m_{\text{1}},\;n_{\text{1}}\right)$ and $\tilde{A_{\text{2}}}=\;\left(l_{\text{2}},\;m_{\text{2}},\;n_{\text{2}}\right)$, as described by Ulutaş et al. [17], are defined as follows:

Addition, $\tilde{A_{\text{1}}}+\;\tilde{A_{\text{2}}}=\;\left(l_{\text{1}}+l_{\text{2}},\;m_{\text{1}}+m_{\text{2}},\;n_{\text{1}}+n_{\text{2}}\right)$
Subtraction, $\tilde{A_{\text{1}}}-\;\tilde{A_{\text{2}}}=\;\left(l_{\text{1}}-l_{\text{2}},\;m_{\text{1}}-m_{\text{2}},\;n_{\text{1}}-n_{\text{2}}\right)$
Multiplication, $\tilde{A_{\text{1}}}\times \;\tilde{A_{\text{2}}}=\;\left(l_{\text{1}}\times l_{\text{2}},\;m_{\text{1}}\times m_{\text{2}},\;n_{\text{1}}\times n_{\text{2}}\right)$
Division, $\tilde{A_{\text{1}}}÷\;\tilde{A_{\text{2}}}=\;\left(l_{\text{1}}÷l_{\text{2}},\;m_{\text{1}}÷m_{\text{2}},\;n_{\text{1}}÷n_{\text{2}}\right)$
Scalar Division (for any positive real number ccc), $\tilde{A_{\text{1}}}÷\;c=\;\left(l_{\text{1}}÷c,\;m_{\text{1}}÷c,\;n_{\text{1}}÷c\right)$

These operations form the mathematical basis for manipulating fuzzy values in the FUCOM-integrated fuzzy-MARCOS methodology employed in this research.

### 2.3. FUSOM integrated fuzzy-MARCOS method

This study implements a hybrid multi-criteria decision-making (MCDM) framework by integrating the FUCOM method with the fuzzy-MARCOS method to evaluate alternatives across multiple criteria. FUCOM, introduced by Pamučar et al. [18], derives consistent and reliable criterion weights using only (n − 1) pairwise assessments, ensuring efficiency and reducing redundancy. It determines the Deviation from Maximum Consistency (DMC) to validate expert input and conserves transitivity in preferences [19]. After determining weights, the fuzzy-MARCOS method assesses alternatives under uncertainty by incorporating fuzzy set theory, addressing unfocused and linguistic data. It comprises matrix normalisation, utility function calculation, and final ranking. The FUCOM–fuzzy-MARCOS integration offers a robust, consistent framework appropriate for complex industrial decision-making scenarios.

Step 1: Construct the aggregated decision matrix under a fuzzy environment.

This matrix was developed by the panel of decision-makers and is represented as follows (Eq. 2):


$$\begin{array}{c} \tilde{D}=[{\tilde{r}}_{ij}{]}_{m\times n} \end{array}$$ (4)

Here, ${\tilde{r}}_{ij}$ denotes the aggregated rating value of the *i*_th_ alternative with respect to the *j*^th^ criterion, calculated using Eq. (3).


$$\begin{array}{c} {\tilde{r}}_{ij}={\prod_{k=\text{1}}^{K}\left({\tilde{r}}_{ijk}\right)}^{\text{1}/K},i=\text{1},\text{2},\text{3}........,m;j=\text{1},\text{2},\text{3}.....n \end{array}$$ (5)

In this equation, ${\tilde{r}}_{ij}$ represents the fuzzy preference rating assigned by the *k*^th^ expert to the *i*^th^ alternative concerning the *j*^th^ criterion. These individual ratings, which form the elements of the matrix defined in Eq. (3), are aggregated using the geometric mean to ensure a consistent and unbiased group evaluation.

Step 2: Expand the Original Matrix to Form the Fuzzy Decision Matrix ($\tilde{D}$)

In this step, the original decision matrix (Eq. 4) is expanded to construct the fuzzy decision matrix by identifying the optimal and worst preference values for each criterion. The ideal solution (*AI*) represents the best performance values, while the anti-ideal solution (*AAI*) reflects the worst-case values across all alternatives.

For benefit (advantageous) criteria, the highest value is considered optimal, whereas the lowest value is deemed anti-optimal. Conversely, for cost criteria, the lowest value signifies the ideal solution, and the highest value indicates the anti-ideal. These extremities are appended to the fuzzy decision matrix to facilitate comparison.

The resulting matrix includes all alternatives *Cm*_*1*_, *Cm*_*2*_... *Cm*_*m*_, evaluated against the criteria *C*_*1*_, *C*_*2*_..., *C*_*n*_ along with the *AI* and *AAI* values, enabling a comprehensive and balanced assessment of all options.


$$\begin{array}{c} \tilde{D}=\begin{array}{c} \begin{array}{cc} & AAI\\ & Cm_{\text{1}}\\ & Cm_{\text{2}} \end{array}\\ \vdots \\ Cm_{m}\\ AI \end{array}\overset{\begin{array}{cccc} C_{\text{1}}\; & \;C_{\text{2}}\; & \;\;\;\cdots \; & \;C_{n} \end{array}}{\left[\begin{array}{cccc} \begin{array}{cc} & {\tilde{r}}_{aai\text{1}}\\ & {\tilde{r}}_{\text{1}\text{1}}\\ & {\tilde{r}}_{\text{2}\text{1}} \end{array} & \begin{array}{cc} & {\tilde{r}}_{aai\text{2}}\\ & {\tilde{r}}_{\text{1}\text{2}}\\ & {\tilde{r}}_{\text{2}\text{2}} \end{array} & \begin{array}{cc} & \cdots \\ & \cdots \\ & \cdots \end{array} & \begin{array}{cc} & {\tilde{r}}_{aain}\\ & {\tilde{r}}_{\text{1}n}\\ & {\tilde{r}}_{\text{2}n} \end{array}\\ \vdots & \vdots & \vdots & \vdots \\ {\tilde{r}}_{m\text{1}} & {\tilde{r}}_{m\text{2}} & \cdots & {\tilde{r}}_{mn}\\ {\tilde{r}}_{ai\text{1}} & {\tilde{r}}_{ai\text{2}} & \cdots & {\tilde{r}}_{ain} \end{array}\right]} \end{array}$$ (6)

Step 3: Generate the Fuzzy Normalised Matrix (${\tilde{n}}_{ij}$)

The fuzzy normalised matrix is constructed using Eq. (5) and (6) to handle cost and benefit criteria, respectively. These equations ensure that all criterion values are scaled consistently, allowing for meaningful comparison across alternatives.


$$\begin{array}{c} {\tilde{n}}_{ij}=\left(n_{ij}^{l},n_{ij}^{m},n_{ij}^{u}\right)=\left(\frac{r_{ij}^{l}}{r_{ai}^{u}},\frac{r_{ij}^{m}}{r_{ai}^{u}},\frac{r_{ij}^{u}}{r_{ai}^{u}}\right) \end{array}$$ (7)

$$\begin{array}{c} {\tilde{n}}_{ij}=\left(n_{ij}^{l},n_{ij}^{m},n_{ij}^{u}\right)=\left(\frac{r_{ai}^{l}}{r_{ij}^{u}},\frac{r_{ai}^{l}}{r_{ij}^{m}},\frac{r_{ai}^{l}}{r_{ij}^{l}}\right) \end{array}$$ (8)

Step 4: Construct the Initial Utility Degree Matrix (*X*)

In this step, the initial utility matrix is developed using elements $x_{ij}$ where $x_{ij}$ > 0, as defined in Eq. (7).


$$\begin{array}{c} X=[\begin{array}{cccc} x_{\text{1}\text{1}} & x_{\text{1}\text{2}} & \cdots & x_{\text{1}m}\\ x_{\text{2}\text{1}} & x_{\text{2}\text{2}} & \cdots & x_{\text{2}m}\\ \vdots & \vdots & \vdots & \vdots \\ x_{n\text{1}} & x_{n\text{2}} & \cdots & x_{mn} \end{array}] \end{array}$$ (9)

Here, *j* = 1,2,3,..., *m*_*j*_ = 1,2,3,...,m represents the alternatives, and *i* = 1,2,3,..., *n*_*i*_ = 1,2,3,..., *n* corresponds to the evaluation criteria.

Step 5: Construct the Normalised Decision Matrix ($n_{ij}^{x}$)

Using Eq. (8), the normalised fuzzy decision matrix is formed, where $n_{ij}^{x}$ denotes the elements of the matrix after normalisation, ensuring comparability across all criteria and alternatives.


$$\begin{array}{c} n_{ij}^{x}=\left\{ \begin{array}{c} @l@\frac{\underset{k}{minx_{kj}}}{x_{ij}}\;\;if\;\;j\in benficial\;criteria\\ \frac{x_{ij}}{\underset{k}{maxx_{kj}}}\;\;if\;\;j\in non-benficial\;criteria \end{array}\right. \end{array}$$ (10)

Step 6: Ranking of Criteria by Experts

Experts are asked to rank the evaluation criteria based on their perceived importance, using their domain knowledge and experience.

Step 7: Determining the Relative Importance Vectors

Vectors representing the relative importance of the criteria are constructed based on the expert rankings. These vectors form the basis for defining the pairwise ratios between adjacent criteria.

Step 8: Formulation of Non-linear Optimization Constraints

To ensure consistency in weight determination, two primary constraints are defined:

Constraint 1 (Pairwise Consistency):

The relative importance between two adjacent criteria is equal to the ratio of their corresponding weight coefficients: $\frac{w_{k}}{w_{k+\text{1}}}=\varphi_{k/(k-\text{1})}$.

Constraint 2 (Transitivity Condition):

The product of consecutive relative importance values must satisfy the transitivity condition: $\varphi_{k/(k+\text{1})}\times \varphi_{\left(k+\text{1})/(k+\text{2}\right)}=\varphi_{k/(k+\text{2})}$.

Step 9: Optimisation Model for Final Weight Determination

A non-linear optimization model is formulated to minimize the deviation from maximum consistency (χ) and determine the final weight coefficients:


$$\begin{array}{cc} & min\chi \\ & s.t\\ & \left|\frac{w_{j(k)}}{w_{j(k+\text{1})}}-\varphi_{\frac{k}{\left(k+\text{1}\right)}}\right|\leq \chi ,\;\forall_{j}\\ & \left|\frac{w_{j(k)}}{w_{j(k+\text{2})}}-\varphi_{\frac{k}{\left(k+\text{1}\right)}}\times \varphi_{\frac{\left(k+\text{1}\right)}{\left(k+\text{2}\right)}}\right|\leq \chi ,\;\forall_{j}\\ & \sum_{j=\text{1}}^{n}w_{j}=\text{1}\\ & w_{j}\geq \text{0},\;\forall_{j} \end{array}$$

Step 10: Final Computation of Criteria Weights

Solving the optimisation model yields the final weight vector: ${\left(w_{\text{1}},\;w_{\text{2}},\;w_{\text{3}},\ldots ,w_{n}\right)}^{T}.$

Step 11: Constructing the Weighted Fuzzy Normalised Decision Matrix (${\tilde{\upsilon }}_{ij}$)

Each element of the fuzzy normalised decision matrix is multiplied by its corresponding criterion weight to generate the weighted fuzzy normalised decision matrix, as defined by Eq. (9).


$$\begin{array}{c} {\tilde{\upsilon }}_{ij}=\left(\upsilon_{ij}^{l},\upsilon_{ij}^{m},\upsilon_{ij}^{u}\right)={\tilde{n}}_{ij}⊗w_{j}=\left(n_{ij}^{l}\times w_{j},n_{ij}^{m}\times w_{j},n_{ij}^{u}\times w_{j}\right) \end{array}$$ (11)

Step 12: Calculating the Aggregate Weighted Values for Each Alternative (${\tilde{S}}_{i}$)

Using Equation (10), the overall weight value for each alternative is computed by summing all the corresponding values in the weighted fuzzy normalised matrix.

Additionally, the total weighted values for both the ideal and anti-ideal solutions are determined in the same manner.


$$\begin{array}{c} {\tilde{S}}_{i}=\sum_{j=\text{1}}^{n}{\tilde{\upsilon }}_{ij} \end{array}$$ (12)

Step 13: Determining the Utility Degree of Each Alternative ($\tilde{K_{i}}$)

Equations (11) and (12) are used to calculate the utility degree of each alternative with respect to the ideal and anti-ideal solutions.


$$\begin{array}{c} {\tilde{K}}_{i}^{-}=\frac{{\tilde{S}}_{i}}{{\tilde{S}}_{aai}}=\left(\frac{s_{i}^{l}}{s_{aai}^{u}},\frac{s_{i}^{m}}{s_{aai}^{m}},\frac{s_{i}^{u}}{s_{aai}^{l}}\right) \end{array}$$ (13)

$$\begin{array}{c} {\tilde{K}}_{i}^{+}=\frac{{\tilde{S}}_{i}}{{\tilde{S}}_{ai}}=\left(\frac{s_{i}^{l}}{s_{ai}^{u}},\frac{s_{i}^{m}}{s_{ai}^{m}},\frac{s_{i}^{u}}{s_{ai}^{l}}\right) \end{array}$$ (14)

Step 14: Computing the Overall Utility Degree

The overall utility degree for each alternative is determined using Eq. (13).

Furthermore, a new fuzzy number $\left(\tilde{d}\right)$is calculated using Eq. (14).


$$\begin{array}{c} {\tilde{t}}_{i}=\left(t_{i}^{l},t_{i}^{m},t_{i}^{u}\right)={\tilde{K}}_{i}^{-}⊕{\tilde{K}}_{i}^{+}=\left(k_{i}^{-l}+k_{i}^{+l},k_{i}^{-m}+k_{i}^{+m},k_{i}^{-u}+k_{i}^{+u}\right) \end{array}$$ (15)

$$\begin{array}{c} \tilde{d}=\left(d^{l},d^{m},d^{u}\right) \end{array}$$ (16)

Here, $d^{l}=\underset{i}{maxt_{i}^{l}},\underset{i}{d^{m}=maxt_{i}^{m}},\underset{i}{d^{u}=maxt_{i}^{u}}$

Step 15: Defuzzification of Fuzzy Utility Values ($df_{crisp}$)

To facilitate comparison, the fuzzy utility values are converted into crisp scores using a suitable defuzzification method, as expressed in Eq. (15).


$$\begin{array}{c} df_{crisp}=\frac{d^{l}+\left(\text{4}\times d^{m}\right)+d^{u}}{\text{6}} \end{array}$$ (17)

Step 16: Utility Function Calculation for Ideal $\left(f\left({\tilde{K}}_{i}^{+}\right)\right)$ and Anti-Ideal Solutions $\left(f\left({\tilde{K}}_{i}^{-}\right)\right)$

Equations (16) and (17) are applied to compute the utility function values corresponding to the ideal and anti-ideal solutions, respectively.


$$\begin{array}{c} f\left({\tilde{K}}_{i}^{+}\right)=\frac{{\tilde{K}}_{i}^{-}}{df_{crisp}}=\left(\frac{k_{i}^{-l}}{df_{crisp}},\frac{k_{i}^{-m}}{df_{crisp}},\frac{k_{i}^{-u}}{df_{crisp}}\right) \end{array}$$ (18)

$$\begin{array}{c} f\left({\tilde{K}}_{i}^{-}\right)=\frac{{\tilde{K}}_{i}^{+}}{df_{crisp}}=\left(\frac{k_{i}^{+l}}{df_{crisp}},\frac{k_{i}^{+m}}{df_{crisp}},\frac{k_{i}^{+u}}{df_{crisp}}\right) \end{array}$$ (19)

Step 17: Final Utility Function and Ranking of Alternatives

Using Eq. (18), the final utility function value for each alternative is obtained.

The defuzzified utility values are denoted as $K_{i}^{-}$, $K_{i}^{+}$, $f\left(K_{i}^{-}\right)$ and $f\left(K_{i}^{+}\right)$ are then used to rank the alternatives, with the alternative having the highest utility function value considered the most preferred choice.


$$\begin{array}{c} f\left(K_{i}\right)=\frac{K_{i}^{+}+K_{i}^{-}}{\text{1}+\frac{\text{1}-f\left(K_{i}^{+}\right)}{f\left(K_{i}^{+}\right)}+\frac{\text{1}-f\left(K_{i}^{-}\right)}{f\left(K_{i}^{-}\right)}} \end{array}$$ (20)

**Fig 2:**
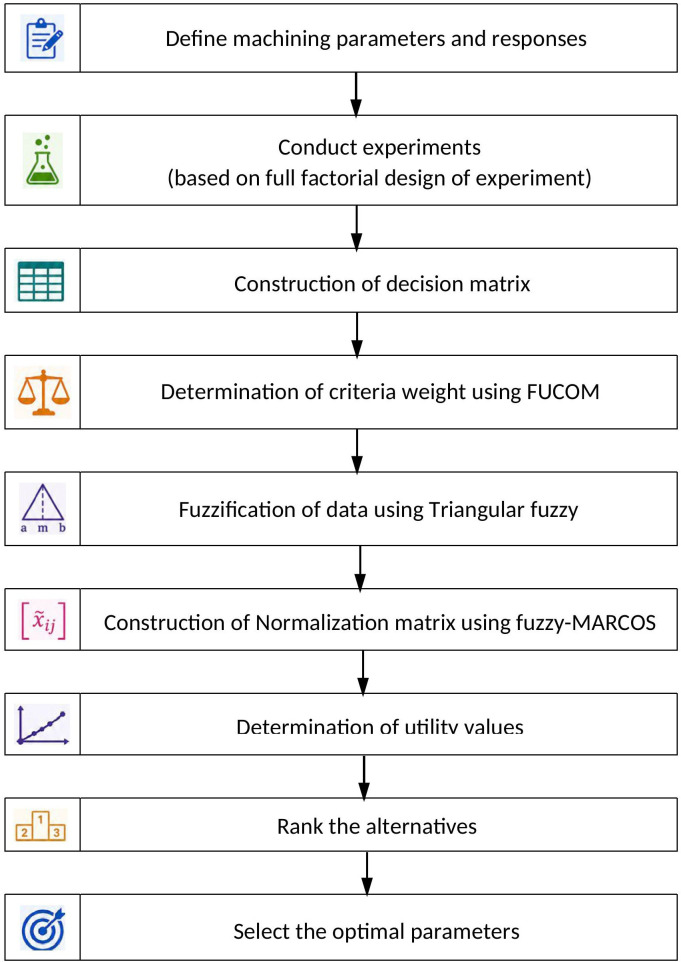
FUCOM fuzzy MARCOS optimisation framework.

A schematic flowchart showing the step-by-step process of the FUCOM–fuzzy MARCOS optimisation method is exhibited in Fig 2 to enhance clarity and understanding.

### 2.4. Ethics Statement

This study does not involve human participants, human data, or animals. The authors performed machining experiments and criterion weighting in the FUCOM method based on their domain expertise and research objectives. No surveys, questionnaires, or interviews were conducted, and no personal or sensitive data were collected. Therefore, ethical approval and informed consent were not required for this study.

## 3. Results and discussion

In this experimental investigation, a full factorial design was employed to develop mathematical models for the key machining parameters, namely depth of cut (*d*_*c*_), feed per tooth (*f*_*t*_), cutting speed (*C*_*s*_), and nano-cutting fluid (*C*_*f*_). The model formulation using a full factorial design was carried out with the help of Minitab 17 statistical software. Table 2 presents the machining conditions along with the corresponding response values obtained. A full factorial design approach was adopted, comprising a total of 27 experimental runs.

**Table 2:** Experimental data.

Experimental No.	Machining process parameters				Machining responses		
	*d*_*c*_	*f*_*t*_	*C*_*s*_	*C*_*f*_	*Ra*	*MRR*	*TWR*
1	2	0.4	775	Oil-Al_2_O_3_	0.492	132.780	0.167
2	2.5	0.3	575	Oil-Al_2_O_3_	1.170	95.730	0.234
3	2	0.2	575	Oil-Al_2_O_3_	0.889	79.830	0.217
4	2	0.3	775	S-Oil	0.872	129.710	0.181
5	2	0.3	675	Oil-Al_2_O_3_	0.681	107.910	0.187
6	1.5	0.3	575	Oil-Al_2_O_3_	0.537	70.110	0.158
7	2	0.3	675	Oil-Al_2_O_3_	0.542	109.180	0.197
8	2	0.3	575	S-Oil	1.230	89.010	0.248
9	2.5	0.3	675	S-Oil	1.270	121.710	0.237
10	2	0.3	675	Oil-Al_2_O_3_	0.632	114.690	0.191
11	2	0.4	675	Water-Al_2_O_3_	0.910	127.950	0.217
12	2	0.2	775	Oil-Al_2_O_3_	0.480	112.340	0.151
13	1.5	0.3	675	S-Oil	1.170	89.870	0.176
14	2.5	0.3	775	Oil-Al_2_O_3_	0.702	132.340	0.159
15	1.5	0.4	675	Oil-Al_2_O_3_	0.670	98.930	0.154
16	2	0.3	775	Water-Al_2_O_3_	0.772	127.250	0.217
17	2	0.2	675	Water-Al_2_O_3_	1.030	105.520	0.202
18	2	0.4	675	S-Oil	1.110	116.780	0.201
19	2	0.2	675	S-Oil	1.080	106.790	0.212
20	2.5	0.4	675	Oil-Al_2_O_3_	0.760	124.930	0.177
21	1.5	0.3	775	Oil-Al_2_O_3_	0.420	109.410	0.148
22	1.5	0.3	675	Water-Al_2_O_3_	0.510	101.830	0.189
23	1.5	0.2	675	Oil-Al_2_O_3_	0.461	85.520	0.134
24	2	0.3	575	Water-Al_2_O_3_	0.953	104.650	0.221
25	2	0.4	575	Oil-Al_2_O_3_	0.740	102.350	0.183
26	2.5	0.3	675	Water-Al_2_O_3_	1.241	119.410	0.207
27	2.5	0.2	675	Oil-Al_2_O_3_	0.997	96.790	0.191

Based on the experimental data, a comprehensive investigation was carried out, as deliberated in the following sections.

### 3.1. Investigation of experimental features

#### 3.1.1. Surface roughness (Ra).

**Fig 3:**
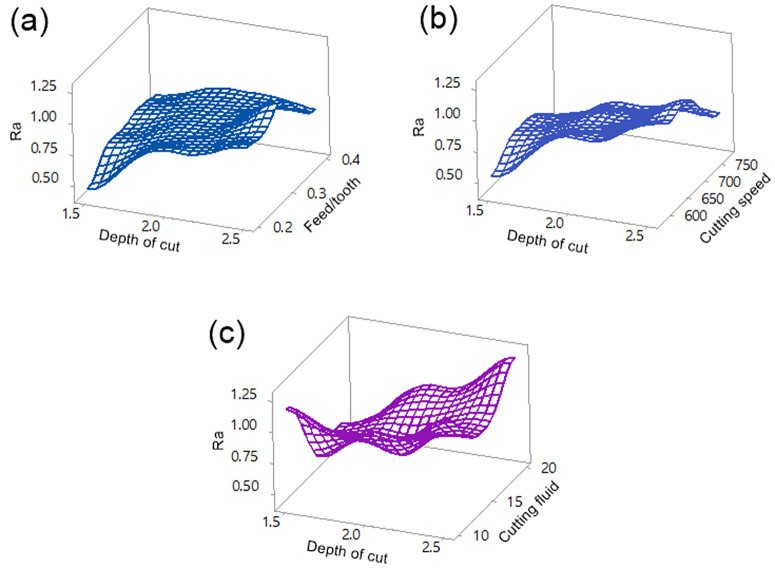
Surface plot for surface roughness (*Ra*) against different machining conditions.

The response surface plots (Fig 3) show a non-linear interaction between surface roughness (Ra) and machining parameters, namely cutting speed and feed rate. Similar non-linear behaviour was reported by Şahin [20] when envisaging surface roughness for mild steel using RSM and ANN models. Initially, rising cutting speed leads to a decrease in *Ra*, attributed to smoother cutting and decreased tool-chip interaction. However, beyond a certain speed, thermal effects, such as micro-burning or tool vibration, can damage the surface finish. Feed rate also strongly affects *Ra*; higher feed rates increase roughness due to more distinct feed marks. The lowest *Ra* values were attained at moderate feed rates and higher cutting speeds. Kar et al. [21] reported a similar effect, noting optimal cutting speed–feed rate combinations minimised *Ra* during CNC milling of Inconel 625.

The surface roughness trend shows a U-shaped response, where initial enhancements from moderate speeds and feeds are offset by worsened finishes at excessive levels due to heat-induced tool wear or chatter. Research on Ti–6Al–4V using PSO and regression showed optimal *Ra* at 100 m/min cutting speed and 0.05 mm/rev feed [22,23], while EN 24 steel studies confirmed collaborative effects in contour plots [24]. This highlights the importance of parameter balance for optimal surface quality.

#### 3.1.2. Material removal rate.

**Fig 4:**
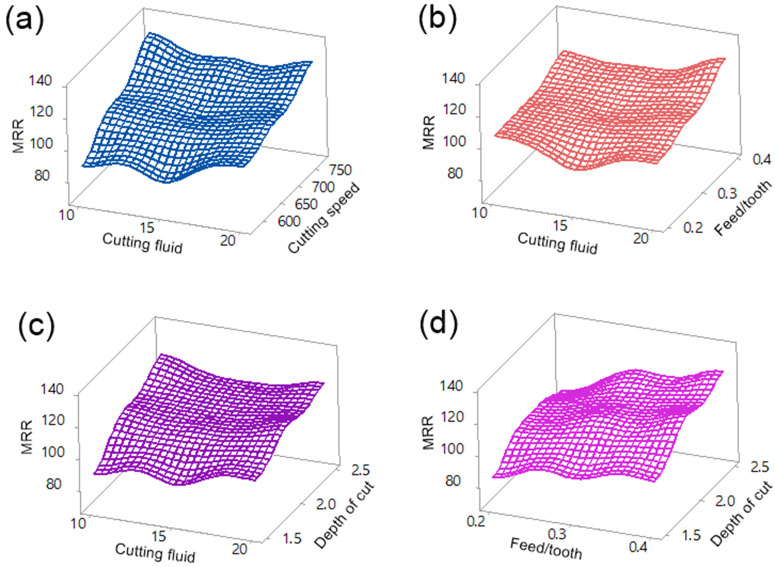
Surface plot for material removal rate (*MRR*) against different machining conditions.

The response surface plots for MRR (Fig 4) show a strong positive association with feed rate and depth of cut, consistent with volumetric removal principles. Increasing these parameters improves MRR, though excessive values can compromise surface integrity and cause tool vibration. A trochoidal milling study supports this, presenting feed rate as the most influential factor (37%), followed by trochoidal step and axial depth [25]. Cutting speed had a reasonable impact, likely due to thermal softening assisting chip formation without proportionally increasing MRR. ANOVA results disclosed depth of cut contributed 72% to MRR, cutting speed 17%, and feed rate 9% [26]. The MRR trend in this study aligned with depth and speed driving performance, while feed rate plays a secondary role. Optimal conditions such as cutting speed ~165 m/min, feed ~0.12 mm/rev, depth ~0.4 mm were identified for EN 24 steel to maximise MRR while maintaining surface quality [6]. Similar insights apply to Ti–6Al–4V machining [23].

#### 3.1.3. Tool wear rate (TWR).

**Fig 5:**
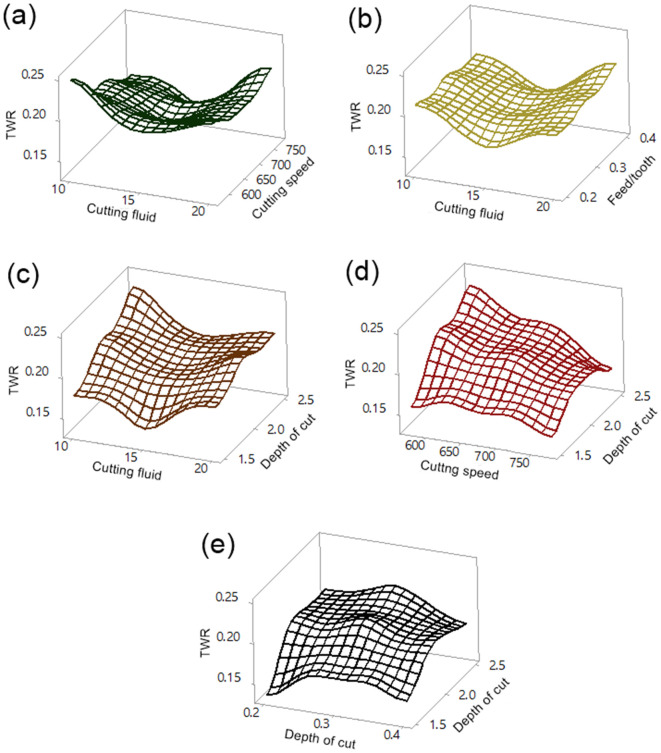
Surface plot for tool wear rate (*TWR*) against different machining conditions.

Tool wear in this study was mainly influenced by cutting speed and feed rate. Raised cutting speeds led to an increase in wear due to higher interface temperatures, endorsing diffusion and adhesion wear mechanisms. Similarly, higher feed rates exaggerated contact pressure and material deformation, accelerating abrasion. Bloul et al. [27] emphasised thermal effects as critical in high-speed ceramic machining. Interestingly, reasonable depths of cut minimised wear by balancing cutting force and tool stability, as designated by a saddle region in the surface plot. The TWR trend (Fig 5) revealed increasing wear with rising speed and feed, while operative cutting fluid application significantly decreased wear. Prasad et al. [28] validated the role of MQL in reducing TWR during Inconel 800 machining. A study using Ti–6Al–4V and Taguchi methods found that TiN-coated tools performed best at 80 m/min and 0.25 mm depth [29]. Moreover, CNN-based models established cutting speed and feed as key factors of wear [30], confirming their importance in tool life optimisation.

### 3.2. Identifies significant group variation using ANOVA

The ANOVA results (Table 3) verified that the regression model for surface roughness is statistically significant (P < 0.001, F = 29.77; Supplementary Table 1). Cutting fluid was the most important factor (P = 0.000, F = 85.76), highlighting its vital role in decreasing surface roughness (*Ra*) through enhanced lubrication and cooling. Significant second-order and interaction effects, such as *C*_*f*_ × *C*_*f*_, *d*_*c*_ × *f*_*t*_, and *d*_*c*_ × *C*_*f*_, further demonstrate the complex relationships affecting surface finish. Subramani et al. [31] similarly testified to the importance of flow conditions on surface quality and tool wear. Interestingly, prime parameters like depth of cut, feed per tooth, and cutting speed showed negligible direct effects (P > 0.90), signifying their impact mainly rises through interactions or quadratic effects. The adequacy of model is supported by a lack-of-fit P-value of 0.642. Marigoudar and Kanakuppi [32] also emphasised that cutting speed, feed, and depth affect surface finish and tool wear.

**Table 3:** ANOVA findings for different machining responses.

Source	Degree of freedom	Sum of squares	Mean squares	F-Value	P-Value
***Surface roughness (Ra)***					
*Regression*	14	1.86681	0.133344	29.770	0.000
*Error*	12	0.05375	0.004479		
*Lack-of-fit*	10	0.04376	0.004376	0.880	0.642
*Pure error*	2	0.00999	0.004995		
*Total*	26	1.92056			
***Material removal rate (MRR)***					
*Regression*	14	6967.73000	497.69500	60.690	0.000
*Error*	12	98.40000	8.20000		
*Lack-of-fit*	10	72.42000	7.24200	0.560	0.784
*Pure error*	2	25.98000	12.99000		
*Total*	26	7066.13000			
***Tool wear rate (TWR)***					
*Regression*	14	14.022176	0.001584	67.600	0.000
*Error*	12	0.000281	0.000023		
*Lack-of-fit*	10	0.000231	0.000023	0.910	0.630
*Pure error*	2	0.000051	0.000025		
*Total*	26	0.022457			

From an experimental perspective, the dominance of cutting fluid as a statistically important factor shows that the selection of a suitable lubrication approach is significant for achieving superior surface quality in industrial machining. The use of nanofluid-based lubrication improves heat dissipation and decreases friction at the tool–workpiece interface, thus minimising surface irregularities and enhancing product finish. This result suggests that, beyond conventional tuning parameters, optimisation of cooling conditions can play a critical role in precision manufacturing.

For material removal rate (MRR), the model is highly important (P < 0.001, F = 60.69), with depth of cut (P = 0.000, F = 22.45) and cutting speed (P = 0.005, F = 11.68) as key contributors. The interaction between cutting speed and cutting fluid (P = 0.008) advises that thermal softening combined with effective lubrication improves chip ejection. Jadhav et al. [33] distinguished similar developments in MRR with increased speed and depth in EN-19 machining. Other important interactions include *d*_*c*_ × *f*_*t*_ and *d*_*c*_ × *C*_*f*_. The quadratic term for depth of cut (P = 0.000) confirms its strong non-linear effect. Maurya et al. [34] also testified collective effects of depth and speed on MRR in EN 36C steel.

The substantial effect of depth of cut and cutting speed on MRR has direct effects on industrial productivity. Increasing the depth of cut significantly improves material removal efficiency, but it must be carefully balanced to prevent excessive tool loading and potential vibration. Similarly, higher cutting speeds can improve machining material due to thermal softening effects but may also introduce thermal damage if not properly controlled. Thus, these parameters must be optimised to achieve maximum productivity without compromising machining stability.

Tool wear was principally influenced by depth of cut (P = 0.000, F = 153.90) and cutting fluid (P = 0.000, F = 103.59) (Supplementary Table 1). Several interactions, namely *C*_*s*_ × *C*_*f*_, *d*_*c*_ × *C*_*s*_, and *f*_*t*_ × *C*_*s*_, were highly significant, reflecting complex thermal and mechanical synergetic effects at the tool–workpiece interface. Maurya et al. [34] confirmed the effectiveness of cutting fluid in decreasing the tool wear rate (TWR) during laser-assisted ceramic machining. Quadratic effects of cutting fluid and depth of cut further suggest non-linear wear behaviour. The robustness of the model is established by a low regression P-value (P = 0.000, F = 67.60) and acceptable lack-of-fit (P = 0.630). Rauf et al. [35] also confirmed significant TWR reduction using MQL during titanium alloy end-milling.

The effective statistical significance of depth of cut and cutting fluid in affecting tool wear highlights their critical role in tool life management. Higher depth of cut raises mechanical and thermal loads on the tool, accelerating wear, whereas efficient cutting fluid mitigates these effects through improved cooling. This suggests that careful control of depth of cut along with the adoption of advanced cooling techniques, such as nanofluids, can substantially extend tool life and reduce machining costs in industrial practice.

### 3.3. Regression analysis of machining responses

**Fig 6:**
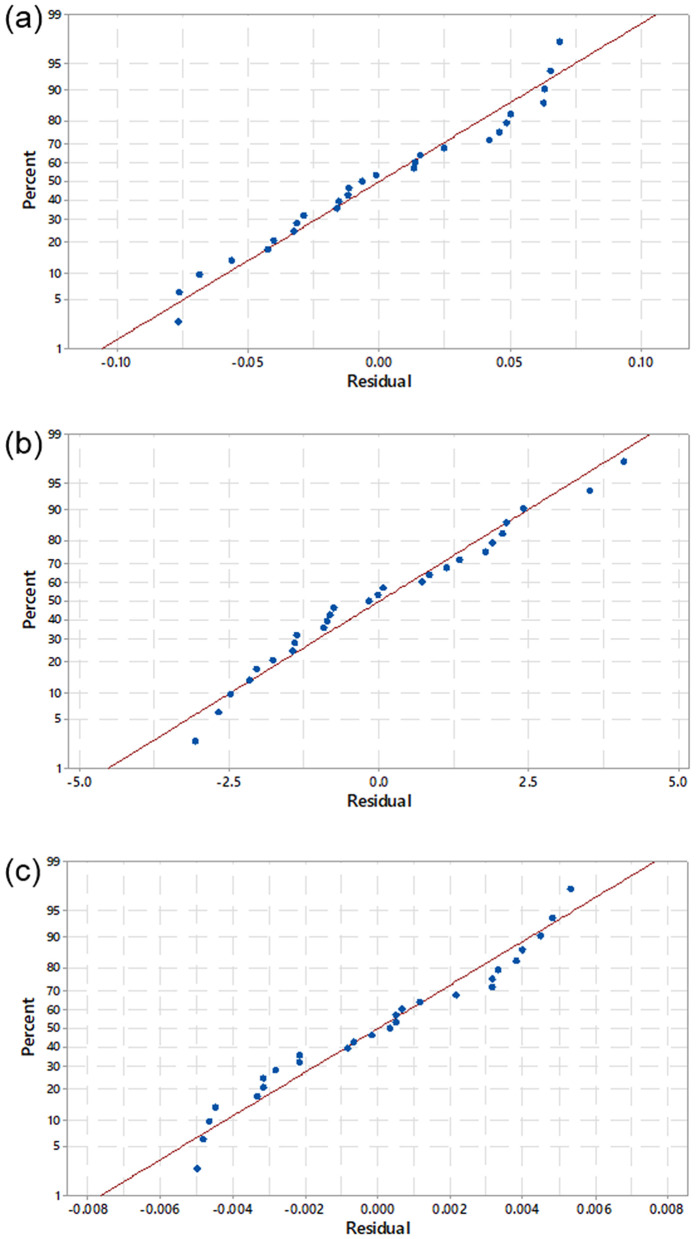
Normal probability plot for (a) surface roughness (b) material removal rate and (c) tool wear rate.


The regression analysis for surface roughness (*Ra*) formed a robust model with an R² of 97.20%, adjusted R² of 93.94%, and predicted R² of 85.71% (Table 4), signifying strong illustrative and predictive competencies. The regression equation classifies cutting fluid as the most significant factor in lowering Ra, confirmed by its large negative coefficient (–0.6132), which replicates improved lubrication and cooling effects. While the linear effects of depth of cut, feed per tooth, and cutting speed were uncertain, their interaction and quadratic terms had a greater effect (Fig 6). Notably, the quadratic term for feed per tooth (+4.05) recommends a threshold beyond which surface quality worsens sharply. Interaction terms like *d*_*c*_ × *f*_*t*_ (–2.227) and *d*_*c*_ × *C*_*f*_ (+0.0631) emphasise the complex mechanisms disturbing surface finish. Similar high accuracies (R² ≈ 96–99%) and findings concerning cutting fluid, feed per tooth, cutting speed, and quadratic effects were testified for AISI 52100 steel using RSM and ANN methods [36,37].

**Table 4:** Model summary for different machining responses.

*S*	*R* ^*2*^	*R*_*(adj)*_^*2*^	*R*_*(pred)*_^*2*^
*Surface roughness (Ra)*			
0.0669265	97.20%	93.94%	85.71%
*Material removal rate (MRR)*			
2.8636100	98.61%	96.98%	93.27%
*Tool wear rate (TWR)*			
0.0048405	98.75%	97.29%	93.58%

$$\begin{array}{cc} \text{Ra }= & \;\text{5}.\text{36 }+\;\text{0}.\text{075 }\times d_{c}+\;\text{0}.\text{23}\times f_{t}\;+\;\text{0}.\text{00014 }\times C_{s}\;-\;\text{0}.\text{6132 }\times C_{f}\;+\;\text{0}.\text{307}\times d_{c}\times d_{c}\;\\ & +\;\text{4}.\text{05 }f_{t}\times f_{t}\;-\;\text{0}.\text{000000 }C_{s}\times C_{s}\;+\;\text{0}.\text{01427 }C_{f}\times C_{f}\;-\;\text{2}.\text{227 }d_{c}\times f_{t}\;-\;\text{0}.\text{001755 }d_{c}\times C_{s}\;\\ & +\;\text{0}.\text{0631 }d_{c}\times C_{f}\;+\;\text{0}.\text{00401 }f_{t}\times C_{s}\;-\;\text{0}.\text{0750 }f_{t}\times C_{f}\;+\;\text{0}.\text{000088 }C_{s}\times C_{f} \end{array}$$

The regression model for material removal rate (MRR) confirmed an excellent fit, with R² = 98.61%, adjusted R² = 96.98%, and predicted R² = 93.27% (Table 4), confirming its robustness. Depth of cut (coefficient = 143.5) and cutting speed (0.640) were the foremost positive contributors, as higher values increase volumetric material removal. However, negative quadratic coefficients for depth of cut (–28.15) and feed per tooth (–155) specify diminishing returns or potential uncertainty at elevated levels. The interaction between depth of cut and feed per tooth (+73.6) was notably important, showing a synergetic effect that can substantially improve productivity when optimised jointly. These conclusions align closely with previous research reporting R² ≈ 98.47% for MRR, emphasising depth of cut, cutting speed, and their interactions as important factors [38,39].


$$\begin{array}{cc} MRR= & -\text{3}\text{5}\text{9}.\text{7}\;+\;\text{1}\text{4}\text{3}.\text{5}\times d_{c}\;-\;\text{1}\text{5}\times f_{t}\;+\;\text{0}.\text{6}\text{4}\text{0}\times C_{s}\;+\;\text{2}.\text{0}\text{0}\times Cf\;-\;\text{2}\text{8}.\text{1}\text{5}\;d_{c}\times d_{c}\;-\;\text{1}\text{5}\text{5}\;f_{t}\times f_{t}\;\\ & -\;\text{0}.\text{0}\text{0}\text{0}\text{2}\text{1}\text{7}\times C_{s}\times C_{s}\;+\;\text{0}.\text{1}\text{8}\text{8}\text{0}\;C_{f}\times C_{f}\;+\;\text{7}\text{3}.\text{6}\;d_{c}\times f_{t}\;-\;\text{0}.\text{0}\text{1}\text{3}\text{5}\;d_{c}\times C_{s}\;-\;\text{1}.\text{4}\text{2}\text{6}\;d_{c}\times C_{f}\;\\ & -\;\text{0}.\text{0}\text{5}\text{2}\;f_{t}\times C_{s}\;+\;\text{6}.\text{2}\text{2}\;f_{t}\times C_{f}\;-\;\text{0}.\text{0}\text{0}\text{9}\text{0}\text{5}\times C_{s}\times C_{f} \end{array}$$

The regression model for tool wear rate (TWR) showed high accuracy with an R² of 98.75%, adjusted R² of 97.29%, and predicted R² of 93.58% (Table 4). Depth of cut appeared as the most significant factor, evidenced by a large positive coefficient (0.6352), as rising cutting depth elevates mechanical load and temperature at the tool–workpiece interface, accelerating wear. On the other hand, cutting fluid and cutting speed had small negative coefficients (–0.04875 and –0.000233, respectively), representing that improved cooling and chip evacuation reduce wear rates. Significant interaction terms, including *C*_*s*_ × *C*_*f*_ and *f*_*t*_ × *C*_*s*_, highlight the combined thermal and mechanical effects on tool degradation. Quadratic effects for feed per tooth (–1.096) and depth of cut (–0.06483) recommend a non-linear, parabolic wear trend, where both very low and very high values are less optimal. These outcomes align with Jadhav et al. [32], who identified depth of cut as the important factor affecting tool wear (P < 0.0001), consistent with the coefficient of the present study. The role of cutting fluid in reducing wear matches the negative coefficient found here. Mohanta et al. [40] also conveyed depth of cut as contributing over 70% to wear, with cutting speed and fluid interactions moderating tool life, supporting the significance of nano-cutting fluid and cutting speed interplay shown in this model.


$$\begin{array}{cc} TWR\;= & \;\text{0}.\text{0}\text{3}\text{5}\;+\;\text{0}.\text{6}\text{3}\text{5}\text{2}\times d_{c}-\text{0}.\text{0}\text{4}\text{8}\times f_{t}-\;\text{0}.\text{0}\text{0}\text{0}\text{2}\text{3}\text{3}\times C_{s}\;-\;\text{0}.\text{0}\text{4}\text{8}\text{7}\text{5}\times Cf\;-\;\text{0}.\text{0}\text{6}\text{4}\text{8}\text{3}\;d_{c}\times d_{c}\;\\ & -\;\text{1}.\text{0}\text{9}\text{6}\;f_{t}\times f_{t}\;-\;\text{0}.\text{0}\text{0}\text{0}\text{0}\text{0}\text{0}\;C_{s}\times C_{s}\;+\;\text{0}.\text{0}\text{0}\text{1}\text{0}\text{7}\text{2}\;C_{f}\times C_{f}\;-\;\text{0}.\text{1}\text{7}\text{0}\text{0}\;d_{c}\times f_{t}\;\\ & -\;\text{0}.\text{0}\text{0}\text{0}\text{3}\text{2}\text{5}\;d_{c}\times C_{s}\;-\;\text{0}.\text{0}\text{0}\text{4}\text{3}\text{0}\text{0}\;d_{c}\times C_{f}\;+\;\text{0}.\text{0}\text{0}\text{1}\text{2}\text{5}\text{0}\;f_{t}\times C_{s}\;+\;\text{0}.\text{0}\text{1}\text{3}\text{0}\text{0}\;f_{t}\times C_{f}\;\\ & +\;\text{0}.\text{0}\text{0}\text{0}\text{0}\text{3}\text{2}\;C_{s}\times C_{f} \end{array}$$

In summary, the regression analysis across all three machining responses, such as Ra, MRR, and TWR, demonstrated that while linear factors such as depth of cut and cutting speed contribute to performance, the combined effects of interactions and non-linear terms are equally critical. Optimisation must consider these complex relationships to balance productivity and tool life.

### 3.4. Optimisation of machining conditions using FUCOM integrated fuzzy-MARCOS algorithm

To address the complexity of optimising multiple machining performance criteria simultaneously, the present study implemented an integrated FUCOM and fuzzy-MARCOS multi-criteria decision-making approach. This hybrid method (as described in section 2.3) effectively incorporated the subjective uncertainties of machining responses, namely surface roughness (Ra), material removal rate (MRR), and tool wear rate (TWR), with fuzzy logic to produce a rational preference ranking for each experimental alternative.

The criteria weights derived using the FUCOM method show typical industrial priorities in machining operations. Surface roughness (Ra) was given the highest weight, as it directly affects surface integrity, dimensional accuracy, and functional performance of machined components, which are significant in tooling and precision applications. The MRR was assigned the second-highest priority, representing the need for improved productivity and lowered machining time in manufacturing environments. The TWR, although important, was given a comparatively lower weight, as tool degradation can be managed through coolant use, tool replacement strategies and planned maintenance. This weighting structure confirms a balanced optimisation method that prioritises product quality while maintaining appropriate productivity and tooling costs, aligning well with practical industrial decision-making. These weights were applied to the normalised fuzzy decision matrix, and the integrated fuzzy-MARCOS framework was used to compute the relative utility scores (fuzzy *K*_*i*_^*⁺*^ values) for all 27 experimental runs (Table 5).

**Table 5:** Utility function values {*f*(*K*_*i*_)} and rank of the alternatives.

Experiment No.	*f*(*K*_*i*_)*	Rank
1	0.00055012	2
2	0.00017239	26
3	0.000198834	24
4	0.000309041	14
5	0.000331713	12
6	0.000365381	9
7	0.000416986	6
8	0.000152594	27
9	0.000199659	23
10	0.000369465	8
11	0.000276123	16
12	0.000529641	3
13	0.000187163	25
14	0.000396066	7
15	0.000345111	10
16	0.000315995	13
17	0.00021911	19
18	0.000225542	17
19	0.000208888	22
20	0.000339249	11
21	0.000612688	1
22	0.000434467	5
23	0.000508654	4
34	0.000223909	18
25	0.000297991	15
26	0.000210137	21
27	0.000215809	20

*Higher values of the utility function, *f*(*K*_*i*_), signify better-performing alternatives and exhibit closer proximity to the ideal solution.

Among the alternatives, Experiment No. 21 (Table 5), which involved a depth of cut of 1.5 mm, feed per tooth of 0.3 mm, cutting speed of 775 m/min, and Oil-Al₂O₃ nanofluid, emerged as the top-ranked configuration. It achieved the highest fuzzy utility score (fuzzy *K*_*i*_^*⁺*^ ≈ 1.0005), demonstrating excellent balance across the three criteria. This alternative achieved minimal surface roughness (~0.42 μm), a high MRR (~109.4 mm³/min), and low TWR (~0.148 mm³/min), marking it as a highly efficient and sustainable machining configuration. Other high-performing alternatives included Experiments 1, 12, and 23, all of which featured combinations of nanofluid-based cooling and moderate-to-low depth of cut settings, which appear to synergistically support lower tool wear and smoother surface finish.

Conversely, alternatives such as Experiment No. 8 and Experiment No. 2 ranked lowest as shown in Table 5, due to a combination of high Ra and TWR and comparatively poor MRR. These configurations typically employed S-Oil or low cutting speeds and feeds, which were less effective in maintaining desirable machining quality. This outcome highlights the essential role of cooling media and parameter synergy in achieving optimal machining responses.

The robustness of the proposed FUCOM-integrated fuzzy-MARCOS framework is further supported by the consistency between the weighted normalised matrix trends and final fuzzy preference values. Unlike traditional deterministic ranking systems, this method offers resilience to input ambiguity and facilitates more nuanced decision-making. Moreover, its adaptability makes it applicable across different materials, tools, fluid combinations and manufacturing objectives.

These findings align with recent literature on hybrid MCDM applications in machining optimisation. For instance, a fuzzy-enhanced MARCOS technique optimises the green machining of SS304 and AISI1045 steels. It validates that integrating fuzzy logic into MARCOS significantly boosts decision insight and properly prioritises criteria across a multi-response problem involving Ra, MRR, and energy consumption [41]. Chowdhury et al. [42] have combined several fuzzy-based MCDM methods, including fuzzy TOPSIS, MOORA, VIKOR, and MARCOS, that applied to the CNC turning of Al 6082-T6 steel. Results show that hybrid fuzzy methods exhibit superior consistency and finer differentiation among close alternatives, validating the precision of fuzzy logic in machining decision-making. Similarly, they have employed multiple fuzzy MCDM methods (CODAS, CoCoSo, MACONT) under q-rung orthopair fuzzy logic to optimise green turning and milling. They confirmed that fuzzy integration improves the reliability and stability of decisions across conflicting objectives like MRR, Ra, energy usage, and tool life. The MOORA, VIKOR, and TOPSIS methods estimate turning with hybrid alumina–graphene nanofluids on AISI 304 steel. The results underscore how nanofluid-based MCDM significantly reduces forces, Ra, and temperature, emphasising both fluid effects and multi-criteria prioritisation with fuzzy-assisted decision-making [43].

The superior ranking of alternatives utilising Al₂O₃-based nanofluids emphasises their substantial engineering advantages in machining applications. Nanofluids improve heat dissipation at the tool–chip interface due to their enhanced thermal conductivity, thus dropping cutting temperature and related thermal damage. Furthermore, the presence of nanoparticles contributes to a rolling and polishing effect, which minimises friction and increases surface finish. These mechanisms collectively result in reduced surface roughness and lower tool wear while continuing a favourable material removal rate. The increased lubrication characteristics of nanofluids also encourage stable cutting conditions, lowering vibration and tool degradation. Hence, the higher ranking of the nanofluid-assisted machining environment in the present study reveals its potential to increase machining efficiency, tool life, and overall process sustainability in industrial applications.

## 4. Conclusions

This study developed a hybrid FUCOM–fuzzy MARCOS method for multi-response optimisation of CNC milling parameters for heat-treated YXR-7 tool steel. The integration of full factorial design, regression, and ANOVA showed important non-linear and interaction effects, with depth of cut and cutting fluid identified as dominant factors. The FUCOM-based weighting selected surface roughness over material removal rate and tool wear, aligning with industrial requirements for surface integrity and productivity. The fuzzy-MARCOS method successfully handled uncertainty and offered a reliable ranking of alternatives, with the optimal condition attaining a constructive combination of low surface roughness (~0.42 µm), high MRR (~109.4 mm^3^/min), and reduced tool wear (~0.148 mm^3^/min).

From an experimental perspective, the proposed method serves as an efficient decision-support tool for selecting machining parameters under inconsistent objectives and uncertain conditions, with potential applicability across various materials and machining conditions. Future work may aim to integrate real-time monitoring, AI-based optimisation, and sustainable machining approaches to further improve its applicability in a smart manufacturing approach.
